# Editorial: From nutrients to nightfall: how metabolism and nutrition interact with sleep and the circadian clock

**DOI:** 10.3389/fnut.2026.1966158

**Published:** 2026-09-02

**Authors:** Ronan Lordan, Omar Ramos-Lopez

**Affiliations:** 1 School of Pharmacy and Biomolecular Sciences, Royal College of Surgeons in Ireland (RCSI) University of Medicine and Health Sciences, Dublin, Ireland; 2 FutureNeuro Research Ireland Centre for Translational Brain Science, Royal College of Surgeons in Ireland (RCSI) University of Medicine and Health Sciences, Dublin, Ireland; 3 Tissue Engineering Research Group (TERG), Royal College of Surgeons in Ireland (RCSI) University of Medicine and Health Sciences, Dublin, Ireland; 4 Medicine and Psychology School, Universidad Autónoma de Baja California, Tijuana, Mexico

**Keywords:** chronobiology, chronometabolism, circadian rhythm, nutrition, sleep

There is growing evidence highlighting the complex interplay between diet, metabolism, sleep, and circadian rhythm, which underscores the importance of not only what we eat but also when we eat. The emerging field of chrononutrition examines how the timing, composition, and quality of food intake interact with circadian biology to influence peripheral clocks, metabolic function, and sleep (1, 2). Conversely, circadian disruption, sleep disturbances, and eating behaviors associated with modern lifestyles, including shift work, social jet lag, and high-fat/high-sugar diets, can impair glucose metabolism, alter appetite, and increase the risk of metabolic, cardiovascular, and sleep-related disorders (3–6).

Despite rapid advances in this field, research remains fragmented across chronobiology, sleep medicine, nutrition, and metabolic physiology. This Research Topic aimed to bring these perspectives together to advance our understanding of how diet, meal timing, sleep, and circadian rhythms interact to shape health and disease. In total, seven contributions were published, covering the areas of metabolism and the circadian clock, eating behaviors and circadian preference, and chrononutrition as a dietary intervention.

Focusing on metabolism, Gipson et al. conducted a randomized crossover study involving 24 adults that were overweight or obese. The authors examined whether the timing of a 24-h fast influences glycemic control and metabolic switching. Afternoon-initiated fasts were found to be associated with lower mean 24-h glucose and lower postprandial glucose responses following a standardized meal. Ketosis was observed after afternoon and evening, but not morning, fasts. Notably, glucose AUC above baseline during the post-fast OGTT was most favorable following morning fasting, indicating that the apparent optimal timing depended on the metabolic outcome being assessed. These findings suggest that the timing of fasting initiation may shape metabolic responses, without any detectable differences in measured insulin or glucagon concentrations and highlight the potential relevance of circadian timing in fasting interventions.

At a population level, Zhang et al. conducted a large longitudinal study of 8,392 middle-aged and older Chinese adults, examining the relationship between insulin resistance and circadian syndrome, integrating metabolic, sleep, and mood-related risk factors. Insulin-resistance surrogate indices were associated with prevalent and incident circadian syndrome in this cohort, with eGDR showing a robust inverse association. Although several indices and the circadian syndrome definition share metabolic components, these findings highlight insulin resistance as an important correlate of broader circadian health and suggest that readily available metabolic indices may help identify individuals at greater risk of circadian syndrome.

Beyond metabolic regulation, circadian timing may also shape everyday eating behaviors and appetite regulation. Chronotype, reflecting individual differences in circadian preference, has increasingly been associated with eating patterns and dietary self-regulation. While the evening chronotype has often been linked to less structured eating patterns, evidence regarding specific behavioral dimensions of eating remains heterogeneous. In a cross-sectional study of 386 adults, Di Rosa et al. investigated the associations between chronotype and eating behavior across BMI categories using the Morningness–Eveningness Questionnaire and the Three-Factor Eating Questionnaire–Revised 18 (TFEQ-R18). In this exploratory cross-sectional sample, morning chronotype was associated with higher cognitive-restraint scores, particularly in selected BMI and sex subgroups, whereas uncontrolled and emotional eating did not differ significantly by chronotype. These findings suggest that circadian preference may be more closely related to the cognitive aspects of dietary self-regulation than to emotional or uncontrolled eating, while underscoring the need for further research in more diverse populations and with consideration of sleep and lifestyle factors.

The relationship between timing and health also extends to women's health. In a cross-sectional study of 426 Japanese women recruited through a mobile health application, Daniel et al. examined the associations between meal timing, sleep timing, chronotype, social jet lag, and symptoms of premenstrual syndrome (PMS) and dysmenorrhea, using multivariable regression analyses. On workdays, later first meals and earlier wake times were associated with greater eating-related PMS symptoms, whereas on free days, earlier first meals, later last meals, longer eating windows, later wake times, and longer sleep duration were associated with greater dysmenorrhea severity. More frequent afternoon snacking was associated with higher PMS symptom scores, while nighttime snacking showed an unadjusted association with moderate-to-severe PMS that did not persist after multiple testing corrections. Taken together, these findings identify meal timing, sleep timing, and snacking behavior as potentially relevant correlates of menstrual symptoms and provide a basis for prospective research in this area.

Several contributions have explored whether modifying the timing of food intake could be used as a practical strategy for improving metabolic and broader health outcomes. A mini-review by Schimenes et al. synthesized evidence on time-restricted eating (TRE), focusing on its effects on body weight, metabolic health, and circadian regulation. Although TRE was associated with modest weight loss and improvements in glycemic control, insulin sensitivity, and lipid profiles in some studies, the review highlighted important inconsistencies and noted that many benefits may be attributable to spontaneous reductions in energy intake. Early and moderate eating windows appeared to be more favorable than later or more extreme approaches, while the limited consideration of chronotype and other circadian factors represents an important gap in the current evidence. In this context, Peters et al. investigated the potential effects of meal timing on sleep in a secondary analysis of a randomized crossover trial involving 31 women that were overweight or obese. Participants completed 2-week periods of early TRE (8 a.m. to 4 p.m.) and late TRE (1 p.m. to 9 p.m.), with sleep being assessed using both objective actigraphy and subjective measures. Early TRE was associated with within-condition improvements from baseline in several actigraphy-derived sleep measures, particularly among participants with poorer baseline sleep. However, changes did not significantly differ between the early- and late-TRE interventions, and subjective sleep measures remained unchanged. These findings suggest that early TRE may be compatible with improvements in objectively measured sleep quality, particularly among individuals with poorer baseline sleep, and warrant further trials designed to directly compare the effects of early and late eating windows.

The functioning of the circadian cycle is also regulated at the genomic level, impacting human physiology (7). Lastly, Grau-del Valle et al. examined changes in circadian gene expression and emotional wellbeing during a 6-month dietary intervention in 50 individuals with obesity. Following a multi-phase intervention, which was associated with an approximate 11% weight loss, participants showed improved metabolic markers, fewer nights of poor sleep, lower state anxiety, and higher PBMC expression of several circadian genes, including *CLOCK, ARNTL, CRY1, DBP*, and *NR1D1*. Although the uncontrolled design and single-time-point gene-expression assessments did not establish the restoration of circadian rhythmicity or a causal effect of weight loss on the molecular clock, the findings provide encouraging evidence that improvements in metabolic status may coincide with changes in clock gene expression and psychological wellbeing. These, therefore, reinforce the close interrelationship between metabolism and the circadian system, where gene expression patterns and emotional issues are involved.

Taken together, these contributions highlight the increasingly interconnected nature of nutrition, metabolism, sleep, behavior, and circadian biology. They also show that it is unlikely that there is a single “optimal” eating schedule: the physiological effects of meal timing may depend on chronotype, metabolic status, sex, genotype, phenotype, sleep patterns, and energy intake. While time-restricted eating remains promising, evidence for benefits beyond calorie restriction is still mixed. The current literature, which includes many cross-sectional and short-term studies, highlights the need for longer, well-controlled interventions that better account for individual circadian characteristics. The role of genetics, epigenetics, the microbiome, and their interaction with circadian rhythms also needs to be considered to gain a holistic view of health status. Ultimately, this Research Topic reminds us that nutrition is not simply about what we eat but when we eat, and that this timing is embedded within the broader rhythms of our lives. Understanding how food, sleep, metabolism, and the circadian clock shape one another may open new avenues for more personalized approaches to health while deepening our understanding of how everyday behaviors influence biology.
